# 498. Baricitinib in Patients with Severe Pneumonia due to COVID-19 in Veracruz, Mexico

**DOI:** 10.1093/ofid/ofab466.697

**Published:** 2021-12-04

**Authors:** Luis Del Carpio-Orantes, Sergio García-Méndez, Gustavo Miguel Zamudio-Severino, Jesús Salvador Sánchez-Díaz, Benito Navarrete-Espinosa, Miguel Ángel Rivera-Viñas, Arturo Salas-González, Andrés aguilar-silva, Sonia Cruz-Albarrán, Jorge Luis Denis-Bravo, Iván Esparza-Mora, Karem Samantha González-Medel, Nelyda Verania Hernández-Zaleta, Edgar Alberto López-Cruz, Laura Guadalupe Montano-Montiel, Jennifer Ivonne Morgado-Hernández, José Alberto Muñoz-Aguilar, Edson Irving Priego-Parra, Reynaldo Reich-Sierra, Noel Jhosimar Sánchez-Jiménez, María Fernanda Tress-Uc, Paola Velázquez-Orozco, Edna Rosario Contreras-Sánchez, Luis Jaime Medrano-Rios, Enrique Ruiz-Castro, Manuel Alejandro Ortiz-Hernández

**Affiliations:** 1 Instituto Mexicano del Seguro Social; Sociedad Mexicana de Virología, Veracruz, Veracruz-Llave, Mexico; 2 Hospital Regional de Alta Especialidad de Oaxaca, Oaxaca, Oaxaca, Mexico; 3 Instituto Mexicano del Seguro Social, Veracruz, Veracruz-Llave, Mexico; 4 Hospital D′María, Veracruz, Veracruz-Llave, Mexico

## Abstract

**Background:**

Patients affected by COVID-19 pneumonia who present severe symptoms with manifest hypoxemia and cytokine storm have a high mortality rate, which is why therapies focused on reducing inflammation and improving lung function have been used, one of them being jakinibs through of the blocking of the JAK tracks.

**Methods:**

Patients who presented data of severe pneumonia due to COVID-19 with data of severe hypoxemia and cytokine storm were selected, from June to August 2020, to whom the SaO_2_/FiO_2_ ratio is measured at the beginning, intermediate and end of treatment, as well as D dimer and serum ferritin. Comorbidity and drugs taken previously are analyzed. The patients being cared for at home.

**Results:**

We included data from 30 patients, 8 (27%) women and 22 (73%) men, with a median age of 58.5 (46.5 - 68.0) years. 23 patients (77%) had comorbidities, the most frequent being arterial hypertension (43%), followed by obesity (30%), type 2 diabetes mellitus (27%), among others. In the laboratory, the medians of D-Dimer 982 ng/mL, Ferritin 1,375 ng/mL and C-Reactive Protein 10.0 mg/dL. Regarding the use of previous medications, we found that 29 (97%) patients had treatment with some medication, the most frequent: azithromycin (77%), ivermectin (53%) and dexamethasone (47%). The median number of medications received was 3. The initial pulse oximetry (SaO_2)_ measurement with room air had a median of 80.5% and the median SaO_2_/FiO_2_ (SAFI) was 134; Regarding the type of SIRA, 90% had moderate SIRA and 10% had severe SIRA.

The median day of evolution on which baricitinib was started was 10 days, all received 4 mg/day, and the median days of treatment with baricitinib was 14.0 days. At follow-up, SaO_2_ at 7 days had a median of 93.0% and the median SAFI at 7 days was 310.0; the median SaO_2_ at 14 days was 95.0% and the median SAFI at 14 days was 452.0. In comparative analysis, baseline SaO_2_/SAFI was significantly lower compared to 7 and 14 days (p = 0.001 for both comparisons). The outcomes, 27 (90%) patients improved and there were 3 (10%) who died.

Demographic Variables

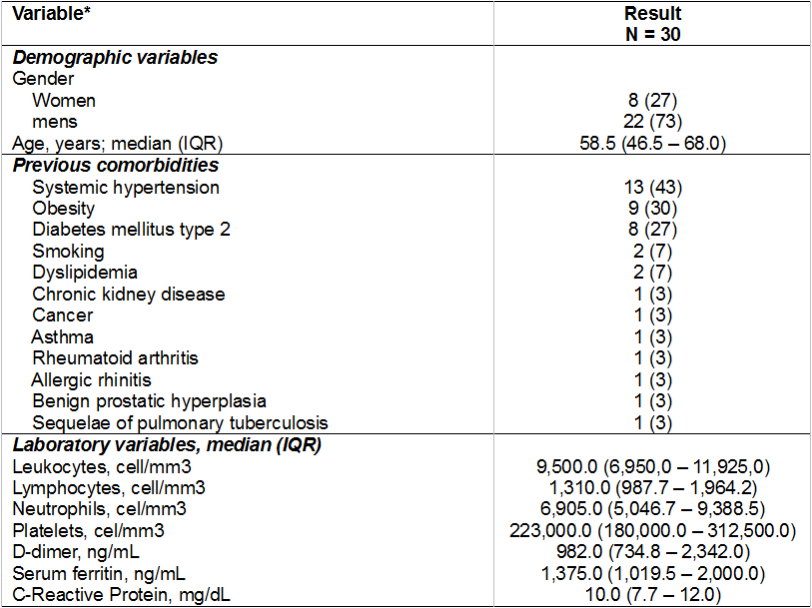

Respiratory Variables

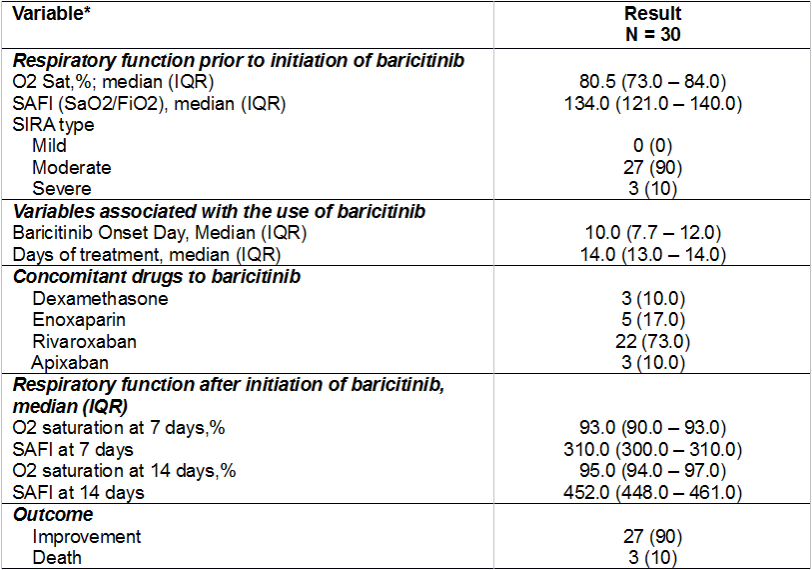

Results on SAFI and SaO_2_

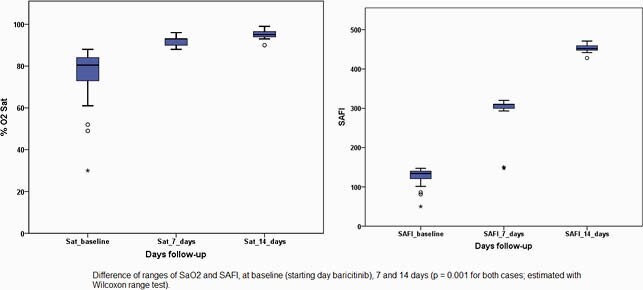

**Conclusion:**

Baricitinib therapy in these patients with severe COVID-19 pneumonia who present with severe hypoxemia and cytokine storm presented good results by improving clinical status and pulmonary failure, with patients being cared for at home and avoiding mechanical ventilation.

**Disclosures:**

**All Authors**: No reported disclosures

